# Supplementary material for: Identification of transformer overload and new energy planning for enterprises based on load forecasting
Source: PLoS One. 2024 Oct 2;19(10):e0311354. doi: 10.1371/journal.pone.0311354 (PMC11446425; doi:10.1371/journal.pone.0311354)
Supplement: Attachment — Submitted filename: PLOS Load forecasting on transformer.pdf [file pone.0311354.s002.pdf]

The paper titled “Identification of transformer overload and new energy planning for enterprises based on load forecasting” is reviewed and I would like to appreciate the authors in presenting the work in a most significant way. But it requires the revisions such as

1. The authors mentioned a proposed overload prediction enterprise methodology for transformers by taking the energy storage system and PV system as examples. Draw and explain your key findings with a load flow diagram and load indication points.
2. The authors stated that your non-linear programming models detect the potential consumers who are under overload. How does this reduce the enterprise's basic tariff and electricity prices and improve efficiency? You did not mention the calculations and descriptions.
3. If a fault occurs in the systems, how does your methodology work? Is there any protection and identification system on the transformer end?
4. English grammar corrections are required, particularly on page 19, 1st paragraph. Check the grammar and technical corrections.
5. Apart from the above-mentioned points, the remaining article is technically sound and explained in a good way.
